# Landscape composition drives winter bird assemblages in agriculture–savanna mosaics of western India

**DOI:** 10.1002/eap.70016

**Published:** 2025-03-06

**Authors:** Tejas Bhagwat, Philippe Rufin, Tobias Kuemmerle, Johannes Kamp

**Affiliations:** ^1^ Department of Conservation Biology University of Göttingen Göttingen Germany; ^2^ Conservation Biogeography Lab, Geography Department Humboldt University Berlin Germany; ^3^ Earth and Life Institute UCLouvain Louvain‐la‐Neuve Belgium; ^4^ Integrative Research Institute on Transformations of Human‐Environment Systems (IRI THESys) Humboldt‐University Berlin Germany

**Keywords:** agriculture–savanna mosaics, annual crops, crop diversity, field size, integrated normalized difference vegetation index, land cover composition and configuration, Palearctic migratory birds, remote‐sensing, savanna grasslands, semi‐perennial crops

## Abstract

Avian biodiversity in agricultural landscapes is declining globally. In Europe and America, agricultural homogenization and the decline of smallholder farming are key drivers of bird population declines. In South Asia, large expanses of compositionally diverse agricultural landscapes still exist. Yet, how resident and migratory avian populations respond to landscape composition and configuration on wintering grounds is largely unknown. Leveraging recent advances in remote sensing, we mapped landscape composition and configuration to analyze their impacts on resident and migratory birds in agriculture–savanna mosaics of western India. We measured landscape composition as the proportional cover of annual crops, semi‐perennial cash crops (primarily sugarcane), savanna and woody vegetation, and compositional heterogeneity as the Shannon diversity of these land cover types. We measured landscape configuration as the mean crop field size. We modeled the abundance and richness of 118 resident and 26 Palearctic migratory bird species as a function of landscape composition and configuration. The richness and abundance of resident birds increased with an increasing land cover diversity and an increasing proportion of semi‐perennial crops. The richness and abundance of Palearctic migratory winter visitors were negatively affected by increasing land cover diversity. A higher proportion of annual crops was associated with higher resident bird densities, whereas the richness response to the proportion of annual crops remained inconclusive. Guild‐based models suggested that migratory carnivores tended to be more abundant in less diverse landscapes with a low proportion of cropland. Open‐ground preferring Palearctic species were positively associated with a higher proportion of semi‐perennial crops and negatively associated with woody vegetation, while shrub‐breeders were positively associated with a high proportion of annual crops and woody vegetation. The effect of mean field size on resident and Palearctic migratory birds was inconclusive. We conclude that (1) winter bird assemblages of resident and migratory species in agriculture–savanna mosaics of western India are driven more by agricultural composition than configuration; (2) resident birds adapt to the high compositional heterogeneity of smallholder agriculture; and (3) Palearctic species primarily rely on compositionally simpler, grassy savannas. Therefore, the maintenance of heterogeneous smallholder agriculture and sparing the savannas from agricultural expansion and afforestation should be key conservation priorities.

## सारांश

शेतजमिनीवर अवलंबून असलेल्या पक्षी वैविध्याचा जागतिक स्तरावर ऱ्हास होत आहे. युरोप आणि अमेरिकेत पिकांचे एकजिनसीकरण (Agricultural homogenization) आणि अल्पभूधारक, मुख्यत्वे उपजीविकेचे साधन म्हणून होणाऱ्या शेतीत (smallholder farming) झालेली घट, ही पक्ष्यांच्या संख्येला मारक ठरत आलेली काही प्रमुख कारणे आहेत. दक्षिण आशियात, शेतीमधील विविधता तुलनेनं मोठ्या प्रमाणावर टिकून आहे. या वैविध्यपूर्ण (composition) शेतजमिनी आणि त्यांची रचना (configuration), स्थानिक आणि स्थलांतरित पक्षी प्रजातींना हिवाळी अधिवास म्हणून किती उपयुक्त ठरतात या बद्दल फार कमी माहिती उपलब्ध आहे. उपग्रहामार्फत मिळणाऱ्या नवीनतम माहितीवर (satellite remote sensing) आधारित, शेतजमिनीवरील विविधता आणि शेतीची संरचना यांचे नकाशे बनवून आम्ही या दोन परिमाणांचा पश्चिम भारतातल्या शेती‐माळरान मिश्रअधिवासातल्या (agriculture–savanna mosaics) स्थानिक आणि स्थलांतरित पक्ष्यांवर काय परिणाम होतो याचा अभ्यास केला. शेतजमिनीवरील विविधता आम्ही त्यावरील बारमाही/नगदी (मुख्यत्वे ऊस) आणि हंगामी पिकांचे प्रमाण, माळरानाचे प्रमाण आणि झाडांचे प्रमाण वापरून शॅनन डायव्हर्सिटी इंडेक्स (Shannon diversity index) च्या आधारे मोजली. शेतजमिनीची रचना आम्ही शेतजमिनीच्या आकाराचे सरासरी क्षेत्रफळ वापरून मोजली. जमीन वापराची प्रमाणे, रचना यांचा वापर करून, आणि पक्ष्यांची विपुलता (abundance) आणि प्रजातींची विविधता (species richness) हे दोन निकष वापरुन आम्ही ११८ स्थानिक आणि २६ स्थलांतरित पक्ष्यांच्या प्रजातींचा या अधिवासातला प्रतिसाद पडताळला. वैविध्यपूर्ण शेतजमिनीवर आणि नगदी पिकांचं जिथे प्रमाण जास्त होतं तिथे स्थानिक पक्षी प्रजातींची विविधता आणि विपुलता जास्त आढळली. स्थलांतरित पक्ष्यांची विपुलता आणि विविधता वैविध्यपूर्ण शेतजमिनीवर कमी होती. हंगामी पिकांचे जास्त प्रमाण स्थानिक पक्ष्यांच्या संख्येला पूरक ठरले पण त्यांचा पक्षीविविधतेवरचा परिणाम निष्कर्षापर्यंत पोहचू शकला नाही. अधिवास आणि खाद्यावर आधारित प्रारूपांनुसार (guild‐based models) समरूप आणि शेतीचं एकूण प्रमाण कमी असलेले अधिवास स्थलांतरित मांसाहारी प्रजातींना पूरक ठरले. बहुतांशी जमिनीवर वावरणाऱ्या प्रजातींना बारमाही/नगदी शेतजमिनी अनुकूल ठरल्या तर झाडांचे प्रमाण जास्त असलेले अधिवास प्रतिकूल ठरले. झुडुपांमध्ये आढळणाऱ्या प्रजातींना हंगामी पिके आणि झाडे जास्त प्रमाणात असलेले अधिवास अनुकूल ठरले. शेतजमिनीच्या सरासरी क्षेत्रफळाचा स्थानिक अथवा स्थलांतरित पक्ष्यांवरचा परिणाम अनिर्णायक राहिला. या माहितीच्या आधारे आम्ही पुढील निष्कर्ष काढले – (१) पश्चिम भारतात हिवाळ्यातील स्थानिक आणि स्थलांतरित पक्ष्यांचे समूह हे शेतजमिनीच्या क्षेत्रफळाऐवजी, शेतजमीन आणि माळराने या अधिवासांशी निगडित विविधतेशी प्रामुख्याने जोडलेले असतात (२) अल्पभूधारक शेतीपद्धती आणि त्यातून निर्माण होणाऱ्या विविध अधिवासांशी स्थानिक पक्षी जास्त प्रभावीपणे जुळवून घेतात आणि (३) स्थलांतरित पक्षी एकसमान, उघडीप असलेले अधिवास, माळराने यावर अवलंबून असतात. परिमाणतः वैविध्यपूर्ण, अल्पभूधारक शेतीची देखभाल, आणि माळरानांना कृषिविस्तारापासून आणि वनरोपणापासून वगळणे, त्यांचे जतन करणे याला जैवविविधता संवर्धनात प्राधान्य द्यायची गरज आहे.

## INTRODUCTION

Globally, avian biodiversity is declining (BirdLife International, 2022; Lees et al., 2022). Agriculture is a major driver of this trend (Green et al., 2005), especially where it expands into natural areas (Foley et al., 2005; Godfray et al., 2010). Additionally, agricultural intensification drives widespread bird population declines through a range of mechanisms (Rigal et al., 2023). This includes shifts towards more capital‐ and labor‐intensive, but also more profitable forms of agriculture, typically associated with higher use of pesticides and fertilizer (Rosenberg et al., 2019). Agricultural intensification often goes hand in hand with mechanization and scale enlargement of agriculture, leading to landscape homogenization and the loss of remaining small habitat patches and structures, thereby leading to biodiversity loss (Fahrig et al., 2015; Stanton et al., 2018), including in birds. Consequently, global bird populations have declined by as much as 25% since the preagricultural era (Gaston et al., 2003). More than half of the bird species in North America and nearly 20% in Europe have experienced rapid declines since the 1970s and 1980s, respectively (Burns et al., 2021; Rosenberg et al., 2019). These declines have been particularly severe in open ecosystems, affecting farmland, grassland, and migratory birds (Gregory et al., 2019; Lees et al., 2022).

Grasslands cover over one‐third of Earth's terrestrial land (Bardgett et al., 2021; Suttie et al., 2005; Wilsey, 2018). For centuries human activities have shaped grasslands; initially through traditional, subsistence agriculture including livestock grazing, hay harvest, and over the past century by intensive agriculture (Bardgett et al., 2021). As grasslands worldwide are increasingly converted into pastures, croplands, and plantations, agricultural developments pose a major threat to their avian biodiversity (Douglas et al., 2023; Food and Agriculture Organization of the United Nations, 2024; Squires et al., 2018). For example, agricultural intensification, habitat loss, and fragmentation of the breeding grounds have driven the decline of songbird populations in the North American prairies (Mahony et al., 2022; Shahan et al., 2017). In South American Pampas, cropland expansions on former grasslands and annual crop cultivation are associated with a lower richness of bird species (Codesido et al., 2008; Schrag et al., 2009). Land cover composition and configuration are crucial factors that decide biodiversity response in agricultural landscapes. Grassland and farmland bird species benefit from mosaic landscapes that include heterogeneous habitats composing seminatural cover, fallows, and diverse crops (Klein et al., 2023; Santana et al., 2017). While such mosaic landscapes are not equal replacements for original grassland habitats, they support higher biodiversity compared with homogeneous agricultural landscapes (Fahrig et al., 2011; Sirami et al., 2019). In the tropics particularly, composition and configurational complexity in agroforestry and traditional livestock systems benefit avian diversity (Velásquez Valencia & Bonilla Gómez, 2019).

Compositionally heterogeneous agricultural landscapes can influence the richness and abundance of grassland bird species based on habitat or diet guild requirements (Hertzog et al., 2023). Across European agricultural landscapes, farmland bird populations benefit from open‐ground patches, hedges, traditional orchards, and grassland patches (Laiolo, 2005; Rösch et al., 2023). The inclusion of foraging crops in agricultural composition and the presence of seminatural cover such as fallows and patches of grasslands surrounding agriculture can have a positive impact on birds of the open land (Frei et al., 2018; Klein et al., 2023; Santana et al., 2017). Likewise, higher configurational complexity can be a significant driver of avian biodiversity (Frank et al., 2024). Smaller field sizes maintain edge habitats and allow access to hedgerows, thus providing crucial nesting and feeding habitats for some farmland birds (Fahrig et al., 2011; Šálek et al., 2018).

While the effects of agricultural intensification on bird populations have been widely studied on their breeding grounds, they are less known for the wintering grounds in the subtropics and tropics where many species spend long periods of their annual migration cycle, especially so in the Asian flyways (Kirby et al., 2008; Somveille et al., 2013; Yong et al., 2021). This is mainly due to the absence of long‐term bird monitoring programs, the lack of accurate, high‐resolution land cover information, and poor recognition and protection of key winter habitats such as grasslands (Gangopadhyay et al., 2022; Ratnam et al., 2016). Especially for the Indian subcontinent, these knowledge gaps are important, given India's major share in the global production of agricultural commodities with over half its land under cultivation (Food and Agriculture Organization of the United Nations, 2017), its large contribution to global bird diversity (Jenkins et al., 2013; Somveille et al., 2013), its significance as the primary wintering ground for Palearctic migratory birds moving in the Central Asian Flyway (Dasgupta et al., 2018; Somveille et al., 2013), and the increasing pressure on biodiversity due to high human population density and land use change (Hinz et al., 2020).

Open ecosystems of India are diverse landscapes that consist of agriculture, grasslands, pastures, and fallow land (Madhusudan & Vanak, 2022; Rawat & Adhikari, 2015). They are also among the most threatened ecosystems in the country. The vast majority of semi‐arid savanna grasslands in India have been labeled as “wastelands” since the colonial era, mainly due to the absence of commercially usable timber in these ecosystems (Ratnam et al., 2016). This forest‐centric conservation legacy persists, as grasslands are frequently earmarked first for agricultural development, infrastructure projects, and afforestation, the latter often with non‐native tree species (Vanak et al., 2014). Agricultural expansion and intensification are characterized by a shift towards monocultures, high‐yielding varieties, and increasing use of fertilizers and pesticides, and have been particularly strong in the open ecosystems of central and western India (Hinz et al., 2020). Consequently, agricultural landscapes are now fragmented and commonly represent agriculture–savanna mosaics (Hinz et al., 2020; Tian et al., 2014). However, links between land use change in agricultural landscapes and the status of biodiversity, including bird populations, are largely unknown.

Over the past two decades, migratory grassland bird populations wintering in India, among them many Palearctic steppe grassland specialists, open‐ground feeders (carnivores and insectivores), and shrub‐dwellers, have experienced sharper declines in abundance compared with resident, especially generalist and wetland species (SoIB, 2020). Palearctic migratory birds that winter in India include species of high conservation concern such as the Endangered Steppe Eagle (*Aquila nipalensis*) and the Critically Endangered Sociable Lapwing (*Vanellus gregarius*). Other open‐ground feeders and shrub‐preferring migratory species such as the Greater Short‐toed Lark (*Calandrella brachydactyla*) and Western Yellow Wagtail (*Motacilla flava*) have also experienced declines (SoIB, 2023). Unlike the Eurasian‐African and Neotropical flyways, there is little information about the trophic response of the migratory grassland birds in the Asian flyways and the relative importance of threats on the wintering grounds, including agricultural change. Closing this knowledge gap is a conservation priority, especially for Palearctic migratory species, as their long‐term survival is determined as much in the wintering range as on the breeding grounds (Bairlein, 2016).

Analyses of the impact of land use change on biodiversity were until recently hindered by the availability of high‐resolution land cover data over large areas. Recent advances in cloud‐based remote sensing now facilitate the delineation of individual crop fields using deep learning (Wang et al., 2022), the mapping of crop types and cropping cycles (Blickensdörfer et al., 2022), and a separation of active from fallow cropland in smallholder systems (Rufin et al., 2022). In India, recent studies have mapped the extent of “Open Natural Ecosystems” (Madhusudan & Vanak, 2022), biomes, vegetation types (Roy et al., 2015, 2006), and agricultural productivity (Dubey et al., 2018; Gangopadhyay et al., 2022; Lee et al., 2022; Rao et al., 2002) using satellite data. However, the available products do not capture seasonal variation in land use, and fail to map crop‐types or delineate single crop fields, mostly due to the small size (on average ca. 1.23 ha) of the landholdings and high crop diversity (Gangopadhyay et al., 2022; Lesiv et al., 2019).

We here aim to (1) map the composition and configuration of agriculture–savanna mosaics at very high resolution and (2) predict the response of bird species richness (total number of species recorded at each point count) and summed abundance (number of individuals across all species recorded at each point count) to the composition and configuration of agricultural and savanna landscapes using a large field dataset. We measured landscape composition as the proportion and diversity of different land use and land cover types, namely annual crops, semi‐perennial crops, woody vegetation cover, and savanna grassland cover. We defined landscape configuration as variation in crop‐field size. We focused on the winter season when bird assemblages in the agriculture–savanna mosaics host resident species, species migrating within the Indian subcontinent, and migratory species from the Palearctic (see Appendix S1: Figures S1–S3). We hypothesized:

1. Landscapes with a higher land cover diversity host a higher resident bird species richness and abundance because compositionally complex landscapes provide spatial and temporal “insurance” (Tscharntke et al., 2012) regarding habitat and food availability.
2. Landscapes with a higher proportion of semi‐perennial crops (especially sugarcane) host a lower resident and Palearctic migratory bird species richness and abundance than those dominated by annual crops because they are compositionally less diverse.
3. Landscapes with a lower proportion of woody vegetation are associated with higher bird species richness and abundance of Palearctic migratory species because these comprise mainly grassland bird species that prefer configurationally simple landscapes, that is, open ecosystems.
4. Landscapes with smaller mean field sizes host a higher resident and Palearctic migratory bird species richness and abundance because configurationally more complex landscapes show more edges (e.g., canals, grassy margins) that provide additional breeding and foraging habitat.

## METHODS

### Study region

**FIGURE 1:**
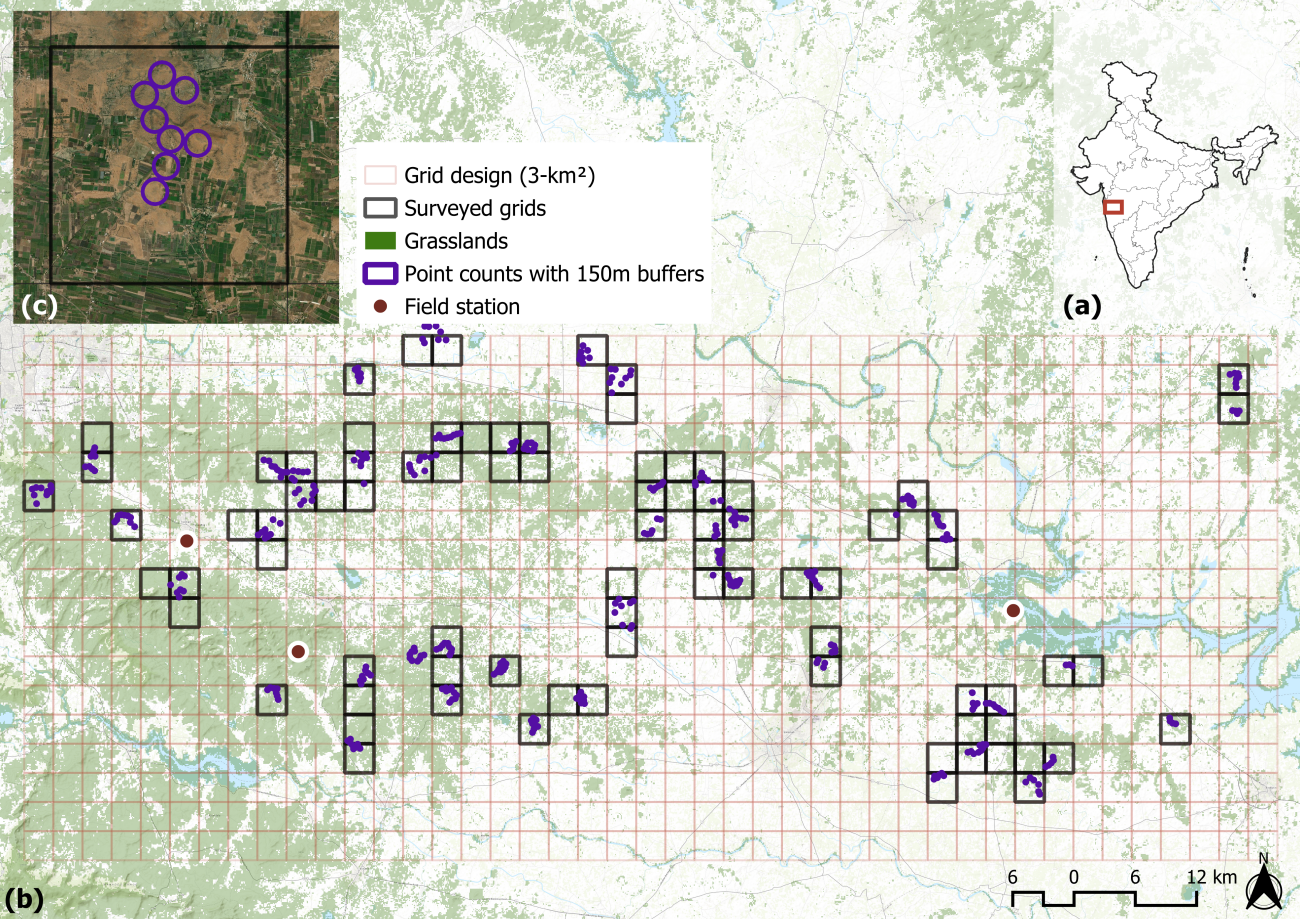
Study region in western India. (a) Administrative boundaries of Indian states and the location of Pune district in Western Maharashtra (red box). (b) Extent of the studied grasslands (copernicus land cover product, green), grid design showing all available 3‐km^2^ grid cells (grid design), accessible grid cells (out of initial 150) that were surveyed with point counts (black), and 371 points (purple) inside or adjacent to each survey site depending on accessibility. (c) Example of a survey site with 150 m buffers around each point for landscape analysis, each point was set apart by a minimum distance of 300 m.

**FIGURE 2:**
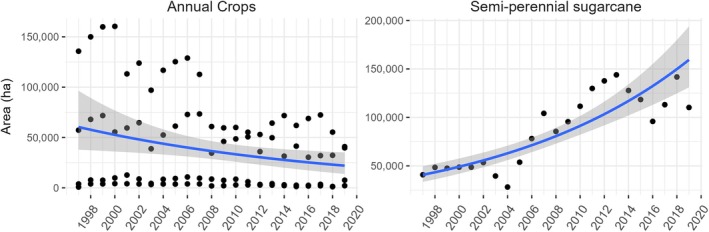
Cultivated area for the annual crops and semi‐perennial sugarcane in Pune district, Maharashtra. The figure shows consistent growth in the area of sugarcane (all three varieties) over the past two decades, mirroring a decline in cultivation areas for traditional annual crop types during the same period. Figure produced by the authors based on the district‐wise, season‐wise crop production statistics from 1997 available from the Open Government Data (OGD) Platform India at https://www.data.gov.in/catalog/district‐wise‐season‐wise‐crop‐production‐statistics‐0; see Appendix S1: Figure S8 for individual crop‐based production area information.

Our study region is situated in the southeast of Pune district in the Indian state of Maharashtra (Figure 1a). Located in the rain shadow of the Western Ghats, the study region receives an annual rainfall of up to 750 mm (Guhathakurta et al., 2020), the majority of which occurs in the months of the Southwest Monsoon (June to September). Natural savanna cover in the region comprises various types of woody plants such as *Terminalia elliptica*, *Shorea robusta*, *Anogeissus latifolia*, and *Tectona grandis* (Ratnam et al., 2019) and native grasses like *Sehima nervosum* and *Dichanthium annulatum* (Rawat & Adhikari, 2015), which are an important grazing resource for the livestock of pastoralist communities (Rawat & Adhikari, 2015). Other nonagricultural vegetation includes invasive shrubs like *Prosopis juliflora* or *Lantana camara*, and other native or non‐native tree plantations planted under afforestation initiatives (Ratnam et al., 2016). Traditional annual crop cultivation in the region follows an asynchronous cycle to optimize the usage of limited water and soil resources. This is achieved through seasonal crop rotation, as grams, groundnut, soybean, sorghum (monsoon), and cotton are cultivated during the monsoon or *Kharif* season, while winter pulses, namely chickpeas, lentils, and pigeon peas, along with sorghum (winter) and onion are cultivated during the winter or *Rabi* season. Sugarcane, a semi‐perennial cash crop, dominates in the region. Three varieties of sugarcane are planted in the region during different times of the year—*Adsali* (June or July), preseasonal (October), and seasonal (December or January) all of which require between 12 and 18 months to complete a single cultivation cycle (Dubey et al., 2018). The sugarcane cultivation area has increased since 1998 at the expense of the annual (*Kharif* and *Rabi*) crop types (Lee et al., 2020; Upreti & Singh, 2017) (Figure 2).

### Bird surveys

We chose our study region and survey design based on a pilot study in winter 2021/2022 with the following objectives: (1) Capture and balance the land use variability in our study region with fragmented savanna grasslands dominating in the west and cropland dominating in the east (Figure 1b) (2) Secure enough available locations for point counts in the study region that have mixed land ownership mainly divided into private land and forest department land with unreliable access or permissions; (3) select survey locations in areas where surveyors were available; and (4) choose survey locations that are logistically reachable and safe in the morning or evening, given the short time windows available for surveys (it gets very hot after 9:00 am and dark by 6:30 pm after which bird activity/visibility drops drastically). Based on the pilot study, we used a grid of 3 km^2^ cells over the study region (Figure 1b) and sampled 150 cells using a stratified‐systematic unaligned sampling method. In total, we were able to access 83 cells and conducted 371 point counts (Figure 1b) in December 2022 and January 2023. This is the period when resident species and migratory birds from the Central Asian Flyway, including Western Siberia, Central Asia, and northern parts of the Indian subcontinent, are present, in addition to the resident Indian species (Appendix S1: Figure S2). In each cell, we conducted point counts at 3–10 point locations, separated by a minimum distance of 300 m (Sutherland et al., 2008) and usually near roads and field borders, as field access was often restricted due to private and government land ownership (Figure 1c). All surveys were carried out during the peak bird activity from 6:30 to 9:30 am and 4:00 to 6:30 pm and only in good weather. The point counts were conducted by 15 experienced local ornithologists and birdwatchers who were familiar with the ecosystems of the study region and the natural history of the bird species. Working in pairs, observers surveyed each point for 5 min. The observers used a snapshot survey technique, a method where the observer records all the birds spotted or heard at one precise, stationary “snapshot” moment during a 5‐min window (Buckland, 2006). The observers used rangefinders to measure the distance of each bird from the observer and only considered the birds within a 150 m radius from the survey point to avoid double counting of birds from the adjacent grid cells or point counts. Since birds were not sensitive to observer presence, no settling period was necessary.

### Land cover mapping

**FIGURE 3:**
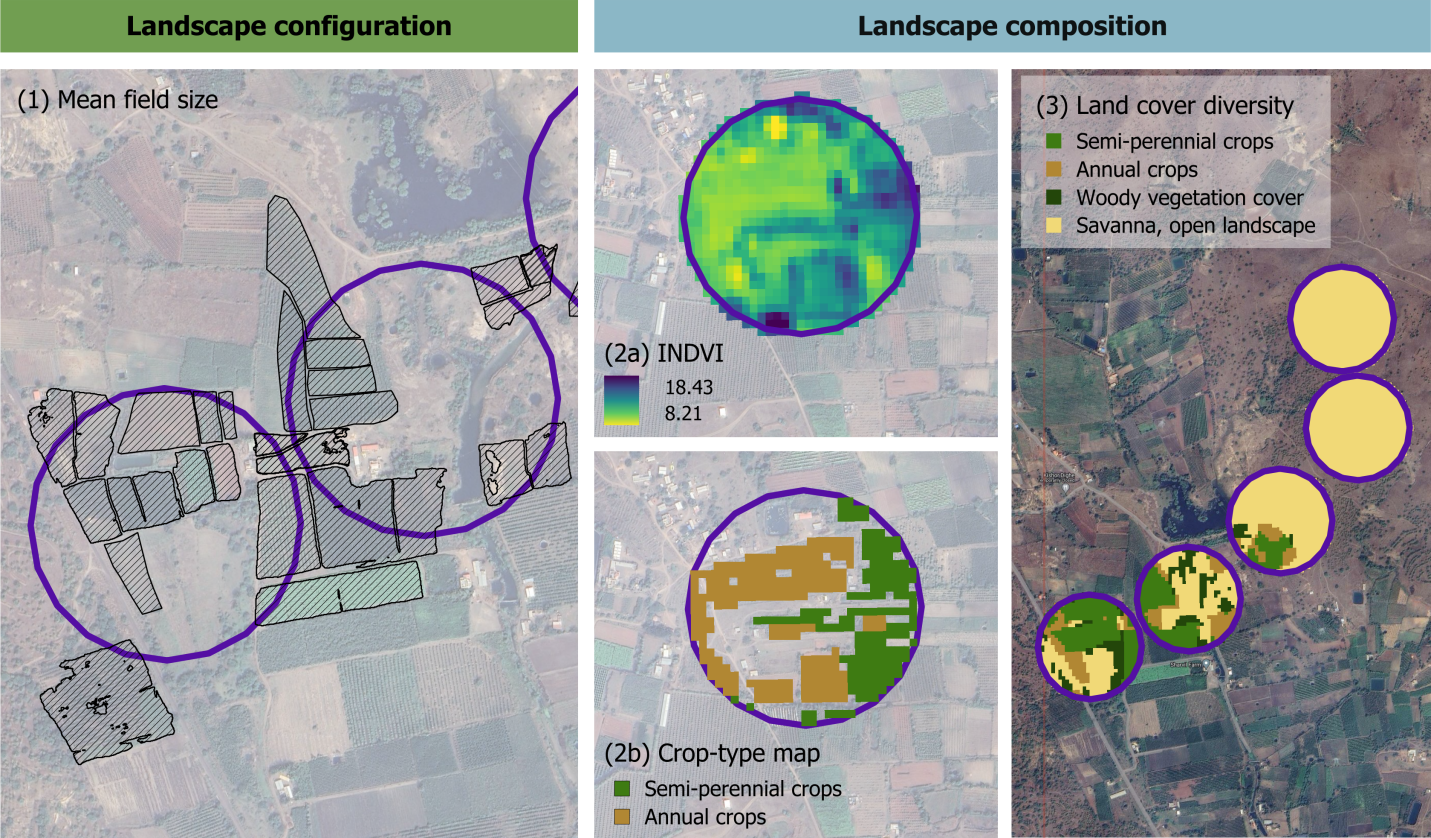
Landscape configuration: (1) Crop fields delineated using Google Earth very high resolution (VHR) imagery. All fields inside the 150‐m buffer and intersecting with the buffer boundary were included in the mean field size calculation. Landscape composition: (2a) Sentinel 2‐based time series of INDVI values during one complete sugarcane cultivation cycle. (2b) Classification of semi‐perennial and annual crops based on an Integrated Normalized Difference Vegetation Index (INDVI) threshold. An identical approach was used on pixels outside crop fields to classify woody vegetation and savanna/open landscape (not shown here). (3) Gradient of land cover diversity illustrated with the 150‐m buffers around five survey points.

We measured *landscape composition* of the landscape as the proportion of (1) semi‐perennial crops (consisting mainly of sugarcane), (2) annual crops (combined *Kharif* and *Rabi*; traditional crop types), (3) savanna grassland cover and (4) woody vegetation cover, inside a 150‐m radius circular buffer surrounding each point count (Figure 3). Additionally, we calculated land cover diversity as a measure of landscape composition for all buffers, using Shannon's diversity index (Figure 3). Since adjacent points were separated by a minimum distance of 300 m, we picked a 150‐m radius while mapping land cover surrounding each point count such that it would not overlap with the adjacent bird survey location. We measured *landscape configuration* using the mean field size for all crop fields that were situated within, or intersecting the border of each 150‐m buffer surrounding the points. For an accurate mean area estimation of the field sizes, we included field boundaries that intersected the 150‐m buffer but were not fully located inside the buffer (Figure 3). We delineated crop fields using a pretrained deep learning model (Rufin et al., 2023; Wang et al., 2022) deployed on Google Earth Pro very high resolution (VHR) imagery. The underlying model architecture is a FracTAL ResUNet specifically designed for crop field delineation (Waldner et al., 2021; Waldner & Diakogiannis, 2020). The model provides multi‐task predictions representing the probabilities of a pixel being a crop field, the probabilities of a pixel being a field boundary, as well as the normalized within‐field distance to the nearest boundary. Model pretraining was conducted with mass data in France and weights were fine‐tuned for use in India using 1.5‐m SPOT data, as described in detail in Wang et al. (2022). We used the pretrained model to produce pixel‐level predictions from Google Earth VHR imagery as described in Rufin et al. (2023) and converted them to individual field instances using hierarchical watershed segmentation. We labeled each segment with a unique ID and removed noncrop field segments such as roads, rivers, and infrastructure manually after visual inspection. A total of 4623 crop fields across the study region were delineated using this method. As there were no land cover maps available to estimate landscape composition, we used remote sensing to produce these data. We first used the “Normalized Difference Vegetation Index (NDVI)” for image pixels completely inside crop fields to measure vegetation productivity. The relationship between vegetation productivity and NDVI is well established (Pettorelli et al., 2011; Tucker et al., 1985) and has been previously applied in sugarcane mapping, yield and crop management analyses (Dubey et al., 2018; Som‐ard et al., 2021). To distinguish between the total vegetation productivity of annual and semi‐perennial crop types, we generated a Sentinel‐2‐based NDVI time series at an interval of 10 days over one complete growing cycle of all sugarcane varieties cultivated in our study region. For each image date, we filled data gaps caused by cloud cover or shadow pixels by using image pixels from an additional 30‐day period both before and after the date. We applied the Savitzky–Golay filter to remove the remaining noise in the time‐series that often results from poor atmospheric conditions or residual cloud pixels (Chen et al., 2004). Finally, we summed the smoothed NDVI values over the complete sugarcane growing cycle to create an Integrated NDVI (INDVI) (see Appendix S1: Figure S4).

Fields planted with sugarcane exhibit higher INDVI values following growth and maturation because of the dense and consistent leaf cover, which remains on site for 12 to 18 months (Dubey et al., 2018; Lee et al., 2022; Som‐ard et al., 2021). In contrast, fields under annual crops for the same period show a lower INDVI during parts of the year as the time series includes fallow periods, harvesting, and periods with low crop cover during germination and early growing stages. Based on these assumptions, we used Otsu's threshold selection method (Otsu, 1979) to perform binary segmentation of INDVI values inside all the 4623 cultivated fields. Otsu's algorithm segments data to maximize inter‐class variance:

$$\sigma_{B}^{2}\left(t\right)=w_{1}\left(t\right)\times w_{2}\left(t\right)\times {\left[\mu_{1}\left(t\right)-\mu_{2}\left(t\right)\right]}^{2},$$

where t is the threshold, $\sigma_{B}^{2}$ is the between‐class variance, $w_{1}\left(t\right)$ and $w_{2}\left(t\right)$ are the probabilities of the two classes divided by the threshold, and $\mu_{1}\left(t\right)$ and $\mu_{2}\left(t\right)$ are the class means. Using this method, we grouped and classified pixels greater than the threshold (i.e., consistently higher productivity and biomass) as semi‐perennial crops (mostly sugarcane), and as annual crops if they were below the threshold value.

To map woody vegetation and grassland cover, we generated a separate Otsu's threshold for the INDVI values of pixels entirely outside the delineated crop fields. Using this new threshold, we classified the “greener” pixels above the threshold (i.e., trees or vegetation with permanent canopy cover) as woody vegetation and pixels with values lower than the threshold as savanna grassland. The processing of Sentinel‐2 data, generation of INDVI, and Otsu's threshold selection method was implemented in Google Earth Engine. These metrics were calculated with the “landscapemetrics” package in R.4.2.2 (Hesselbarth et al., 2019).

### Ground truthing and classification accuracy assessment

We ground truthed delineations of annual crops and semi‐perennial crops. During the bird surveys, we recorded the crops surrounding each point. In the case of multiple crops, we recorded the most dominant crop at the site. Using this information together with very high‐resolution Google Earth imagery, we manually digitized crop field segments inside 150‐m buffers and labeled fields as semi‐perennial crops if they were sugarcane and as annual crops if they were *Rabi* crops such as sorghum, wheat, onions, groundnut, pigeon peas, winter wheat, guava, pomegranates, and custard apple. To ground truth the classification of seminatural land cover features, namely woody vegetation and savanna grassland, we created validation polygons using Google Earth imagery and land cover classification of India's nonforested Open Natural Ecosystems (Madhusudan & Vanak, 2022). We combined all four land cover categories in the final ground truth data and sampled 1500 pixels using stratified weighted random sampling, where the allocation of samples across the classes was proportional to the area of each predicted land cover class. We compared class assignments and predicted land cover at the selected 1500 pixels and generated a confusion matrix (see Appendix S1: Table S1).

### Models of bird species richness and abundance

We modeled bird species richness and abundance as functions of the proportion of semi‐perennial crops, the proportion of annual crops, the proportion of woody vegetation, land cover diversity, and mean field size. We first modeled bird species richness and abundance responses for resident species of the Indian subcontinent (*n* = 118). Next, we fitted models for bird species richness and abundance of Palearctic migratory birds that winter in India (*n* = 26). Additionally, we fitted three guild‐specific abundance models to assess the trophic response of the Palearctic migratory birds. We distinguished carnivores (mostly raptors such as kestrels and harriers), open‐ground feeders (e.g., larks), and species preferring shrub habitats on the breeding grounds.

We fitted Bayesian Generalized Linear Mixed Effects Models (GLMM) using the “rstanarm” package in R.4.2.2 (Goodrich et al., 2024). We standardized (*Z*‐scores) all environmental variables prior to modeling and checked for multicollinearity between all covariates using Spearman's correlation coefficient with a cutoff point of |*r*| < 0.5. Consequently, the proportion of savanna grassland cover was not used as a predictor, as it was significantly correlated with the areas of annual (*r* = −0.59) and semi‐perennial (*r* = −0.78) cultivation (see Appendix S1: Figure S5). We used weakly informative priors drawn from a normal distribution with a mean of 1 and an SD of 2.5. We evaluated the convergence of Markov chains using the Gelman–Rubin statistic (Gelman & Rubin, 1992).

Model coefficients were interpreted as effect sizes, and effects were deemed credible with “high certainty” if their 95% credibility interval did not include zero and with “low certainty” if their 50% credibility interval did not include zero. We considered 50% intervals in our methods for two reasons. First, this approach accounts for effects that are less pronounced but show a clear positive or negative tendency (Amrhein et al., 2019; McShane & Gelman, 2022; Pflüger et al., 2024). Second, avoiding a rigid statistical threshold allows for a more transparent and nuanced interpretation of our results and discourages selective reporting for significant outcomes (Amrhein et al., 2019; Gelman, 2016). Effects were deemed inconclusive if both 95% and 50% credible intervals included zero. We assessed our models using graphical posterior predictive checks produced with the “bayesplot” package (Gabry et al., 2019). Using this setup, we built separate models for (1) resident bird species richness and abundance comprising 118 species recorded, (2) abundance and species richness of the Palearctic migratory bird species, and (3) guild‐specific abundance of the Palearctic migratory bird species that were grouped into three habitat and diet guilds, namely carnivores, species preferring open ground, and species preferring shrubs.

For a given survey point, we accounted for the variation in bird response *C* for a given survey point *i* by assuming it to be a Poisson process:

$$C_{i}\sim \text{Poisson}\left(\lambda_{i}\right),$$

where $C_{i}$ is the vector of possible bird responses (abundance, richness, in {0,1,2,3,…, *N*}) at point *i*, and $\lambda_{i}$ are the expected mean values of the bird response. We fitted the following model using the generic formula:

$$\begin{array}{l} \;\log\left(\lambda_{0}\right)=\alpha_{0,i}+\beta_{1}\left(\text{proportion of semi}-\text{perennial crops}\;\right)\\ \;+\beta_{2}\left(\text{mean field size}\right)\\ \;+\beta_{3}\left(\text{proportion of woody vegetation cover}\right)\\ \;+\beta_{4}\left(\text{proportion of annual crops}\right)\\ \;+\beta_{5}\left(\text{Shanno}n^{\prime }s\;\text{diversity index}\;(\text{land cover diversity}\right))\\ \;+\beta_{6}\left(\text{mean elevation}\right)+\beta_{7}\left(\text{built}-\mathrm{up}\right), \end{array}$$

where $\alpha_{0,i}$ are the varying regression intercepts as random sample point effects defined to be drawn from a normal distribution with a mean $\mu_{\alpha }$ and variance ${\sigma^{2}}_{\alpha }$ as estimated hyper‐parameters:

$$\alpha_{0,i}\sim \text{Normal}\left(\mu_{\alpha },{\sigma^{2}}_{\alpha }\right).$$

We added elevation and the proportion of built‐up (e.g., infrastructural developments) as control covariates, using data from the Open Buildings dataset (v3) (Sirko et al., 2021). This was done because there was a tendency for grasslands to be situated at higher elevations, and because synanthropic bird species might reach higher diversity and abundance in areas with more human habitation.

## RESULTS

We used a Markov Chain Monte Carlo (MCMC) algorithm to derive posterior distributions from four chains, each consisting of 6000 iterations with a burn‐in of 2000 iterations which were sufficient to achieve convergence ($\hat{R}<1.1$). All models had a good fit. For posterior predictive distributions see Appendix S1: Figures S6 and S7.

**FIGURE 4:**
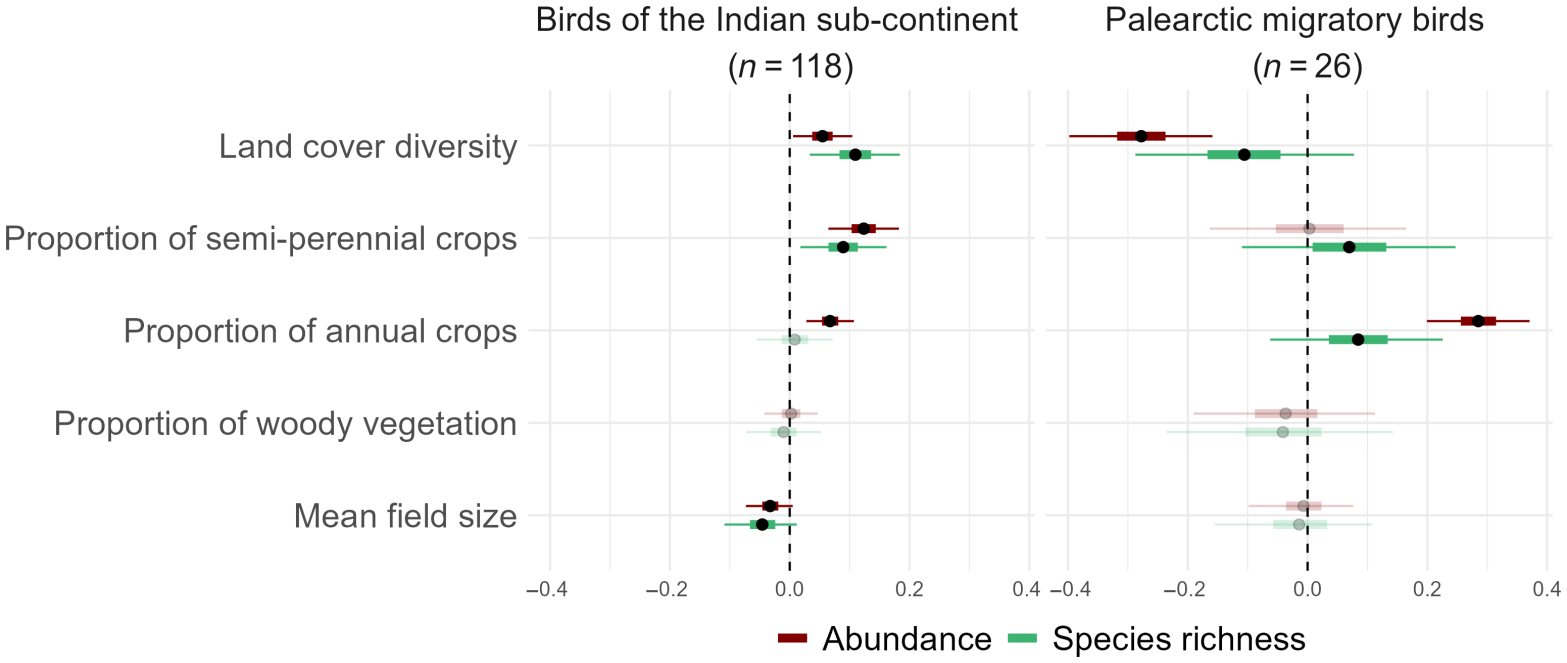
Effects of landscape composition and configuration on resident bird species (*n* = 118) richness and abundance (left panel), and species richness and abundance of Palearctic migratory species (*n* = 26) wintering in the study region (right panel). Black dots show estimated coefficients (posterior means), thick lines show their 50% credible interval and thin lines show the 95% credible interval from Bayesian Generalized Linear Mixed Models. Inconclusive effects are plotted partly transparent.

**FIGURE 5:**
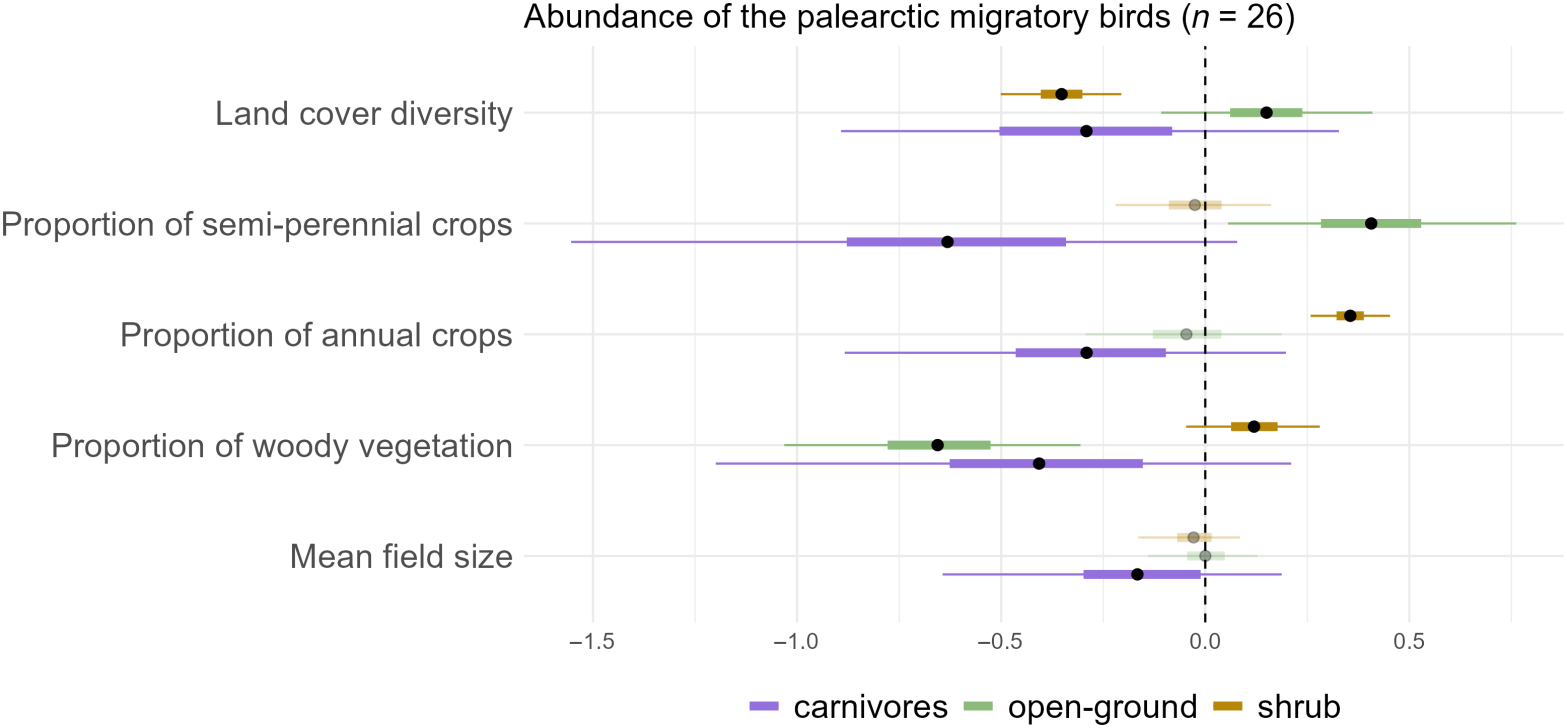
Effects of landscape composition and configuration on the abundance of Palearctic migratory bird guilds—carnivores, species preferring open ground, and species preferring shrubs. Black dots show estimated coefficients (posterior means), thick lines show their 50% credible interval and thin lines show the 95% credible interval from Generalized Linear Mixed Models.

A high proportion of land cover diversity had a strong, positive effect on resident (*n* = 118) species richness (high certainty, β = 0.110, 95% CI = 0.034, 0.184) and abundance (high certainty, β = 0.055, 95% CI = 0.006, 0.104) (Figure 4), and a strong negative effect on the abundance of Palearctic migratory (*n* = 26) species (high certainty, β = −0.278, 95% CI = −0.398, −0.159), especially on species preferring shrubs (high certainty, β = −0.352, 95% CI = −0.501, −0.206) (Figure 5). A high abundance of both resident (high certainty, β = 0.067, 95% CI = 0.028, 0.107) and Palearctic migratory (high certainty, β = 0.285, 95% CI = 0.199, 0.371) was associated with a high amount of annual crops (Figure 4), especially in shrub‐inhabiting migrants (high certainty, β = 0.355, 95% CI = 0.258, 0.453) (Figure 5).

A high proportion of semi‐perennial crops had a positive effect on resident species richness (high certainty, β = 0.089, 95% CI = 0.018, 0.162) and abundance (high certainty, β = 0.124, 95% CI = 0.065, 0.182) (Figure 4). Among long‐distance migratory species, open‐ground feeders benefited from a high proportion of semi‐perennial crops (high certainty, β = 0.407, 95% CI = 0.056, 0.762) while the abundance of carnivorous species was negatively affected (low certainty, β = −0.632, 95% CI = −1.554, 0.078).

The effect of a high amount of woody vegetation on overall bird species richness and abundance was inconclusive for both resident and Palearctic migratory species. However, our guild‐based abundance models for Palearctic migratory species indicated that their abundance was likely to be driven by the habitat and diet of the migratory species (Figure 5). The abundance of species preferring open ground (high certainty, β = −0.656, 95% CI = −1.031, −0.306) and carnivores (low certainty, β = −0.407, 95% CI = −1.199, 0.211) declined with an increase in woody vegetation, while that of shrub dwellers tended to increase (low certainty, β = 0.120, 95% CI = −0.047, 0.281) from it (Figure 5).

A high mean field size had a negative but rather weak effect on resident species richness (low certainty, β = −0.046, 95% CI = −0.109, 0.012) and abundance (low certainty, β = −0.033, 95% CI = −0.073, 0.005). The effect of mean field size was inconclusive for Palearctic migratory species, with a weak negative tendency for the abundance of carnivorous migratory species (low certainty, β = −0.166, 95% CI = −0.644, 0.187) (Figure 4).

## DISCUSSION

Globally, grassland ecosystems are threatened by agricultural intensification and expansion. Both processes are major drivers of the decline in grassland avifauna (Douglas et al., 2023). Structural and compositional complexity in agricultural landscapes characterized by a high amount of seminatural habitat, diverse crops, and small field sizes can play a significant role in halting and reversing these declines (Fahrig et al., 2015; Sirami et al., 2019). However, most of the evidence for the impacts of agriculture on bird population trends comes from the breeding grounds in Europe and North America, regions with predominantly mechanized and homogenized agriculture.

We provide novel insights into the compositional and configurational drivers of the richness and abundance of resident and Palearctic migratory species on the wintering grounds, here a mosaic of smallholder agricultural landscapes and savannas of South Asia. Our results reveal that compositionally more complex agriculture–savanna mosaics host a higher richness of resident species at higher abundances, but fewer Palearctic bird species at lower abundances compared with more open savannas. Resident species were also more associated with semi‐perennial crops such as sugarcane than Palearctic migratory species. The response of Palearctic migratory species to the composition of the agriculture–savanna mosaic was highly guild‐specific, that is, related to habitat preference and feeding ecology. Our results suggest that composition plays a more significant role in the distribution of the Palearctic migratory species on subcontinent wintering grounds than configuration.

Over the last two decades, Palearctic migratory species in India have declined by over 50% while the populations of resident species have remained largely stable (SoIB, 2023). Land use/land cover change drivers of these disparate trends in bird populations are poorly understood. We show that the resident bird species richness in agriculture–savanna mosaics is especially high during the winter season, where landscapes are compositionally complex, that is, characterized by varying proportions of smallholder cultivation (annual and semi‐perennial), woody vegetation, and savannas. This supports our first expectation. As “diversity begets diversity” (Stein et al., 2014), resident species richness might primarily be driven by a high diversity in foraging niches, including invertebrates and small mammals on the open ground, grains from the standing crops, and fruiting trees, which collectively benefit multiple species.

We expected higher species richness and abundance in agriculture–savanna mosaics with higher annual crop cover compared with landscapes with a higher semi‐perennial crop cover (often sugarcane). Our results back up our expectation regarding the abundance of resident and Palearctic migratory species with an increase in annual crop cover. Typical annual winter (*Rabi*) crop composition in our study region comprises winter sorghum, maize, ground nut, gram (chickpeas), Arhar (pigeon peas), winter wheat, onion, safflower, millets, sunflower, mustard and sesame (per “District‐wise, season‐wise crop production statistics from 1997” available from the Open Government Data (OGD) Platform India at https://www.data.gov.in/catalog/district‐wise‐season‐wise‐crop‐production‐statistics‐0). Annual winter crops are sowed between September and January, thus offering a heterogeneous agriculture–savanna mosaic comprising fallow land, crops with varying vegetation structure, and a variety of food resources. This maintains a compositionally more complex landscape through the winter compared with areas with sugarcane cultivation. Our results show that both resident and Palearctic migratory birds are abundant in landscapes with a high proportion of annual crops, especially migratory shrub‐preferring species such as Siberian stonechat (*Saxicola maurus*), Red‐headed bunting (*Emberiza bruniceps*) and Booted warbler (*Iduna caligata*), which were frequently recorded during the surveys and likely benefit from annual crop diversity.

Our results suggest an increase in species richness and abundance of resident species with an increase in semi‐perennial crop cover in our study region. For Palearctic migratory species, the effect was strongly positive for the abundance of open‐ground species. Semi‐perennial sugarcane is heavily irrigated during the winter season. Irrigation channels and other water bodies surrounding the sugarcane are rich in food sources including fish, vertebrates, and regional water plants where waders and wetland species often forage (Mathialagan et al., 2022). Similarly, passerines find refuge and foraging habitats along uncultivated edge vegetation bordering sugarcane fields (Miller et al., 2011). This could explain the positive response of the resident bird populations and migratory open‐ground species in semi‐perennial agriculture as our data included resident species benefiting from the presence of water such as the lapwings (*Vanellus indicus*, *Vanellus malabaricus*), kingfishers (*Halcyon smyrnensis*), as well as resident and migratory passerines such as babblers (*Argya striata*, *Argya malcomi*), short‐toed lark (*Calendrella brachydactyla*), tawny pipit (*Anthus campestris*) and wagtails (*Motacilla flava, Motacilla cinerea*) which feed on open ground (see Appendix S1: Figure S2).

At the same time, our results reveal that the abundance of Palearctic birds of prey declines with an increase in semi‐perennial crops. This aligns with other findings on Palearctic migratory species in India, especially grassland species, which primarily rely on homogeneous open ecosystems. For instance, harrier (*Circus* spp.) populations have experienced sharp declines in southern India following the rapid development of infrastructure, invasion of *Prosopis juliflora*, and other woody vegetation on former savannas that provide optimal roosting and foraging habitats (Ganesh & Prashanth, 2018; Saravanan et al., 2021). In our study region, the negative impact of semi‐perennial sugarcane highlights the ecological importance of compositionally simpler savannas for migratory carnivores, namely the Pallid harrier (*Circus macrourus*), the Endangered Steppe eagle (*Aquila nipalensis*) and the Common kestrel (*Falco tinnunculus*), which were most frequently recorded in our data and hunt and roost exclusively in these habitats (Kher & Dutta, 2021). Our expectations regarding semi‐perennial crop cover were therefore partly met.

Landscape configuration is a key driver of avian biodiversity in agro‐ecosystems (Macchi et al., 2020; Marcacci et al., 2022). On the breeding grounds, smaller field sizes often benefit avian biodiversity in agricultural landscapes, as they provide better access to field boundary habitats, hedgerows with suitable habitat niches, and food resources (Fahrig et al., 2015; Noack et al., 2022; Šálek et al., 2021; Vickery et al., 2009). In landscapes dominated by intensive agriculture, other land cover features such as woody vegetation can mitigate the negative effects of increasing field size (Frank et al., 2024). Outside the breeding grounds, configurational complexity added by seminatural habitats, woody vegetation, and shrubs can be a greater determinant for avian biodiversity than field size (Marcacci et al., 2022; Mellink et al., 2017). This might be because the gradient in field size is much smaller on the wintering grounds in sub‐Saharan Africa and South Asia, where small and very small fields dominate compared with the breeding grounds in the Northern Hemisphere (Fritz et al., 2015). So far, the effects of landscape configuration on avian communities are rarely addressed for wintering grounds in South Asia, even less so for Palearctic migratory birds.

Our results reveal that an increase in woody vegetation cover significantly reduces the abundance of Palearctic birds preferring open ground, negatively impacts the abundance of carnivorous species, and benefits species preferring shrubs. This is consistent with our expectation regarding the abundance of Palearctic migratory species in our data which primarily consists of steppe specialists. Here, negative impacts on the carnivores and open‐ground species may be explained by the fragmentation and loss of uncultivated savannas due to afforestation initiatives (National Mission for a Green India, 2024) and invasion of *Lantana camara* and *Prospis juliflora*, which were commonly observed during our surveys and contribute significantly to grassland degradation in India (Kannan et al., 2013; Ratnam et al., 2016; Vanak et al., 2014). In contrast, smaller field size only had a marginal and low certainty effect on the species richness and abundance of resident or Palearctic migratory species. Despite very high yields of sugarcane, the average field size for annual and semi‐perennial crops in India remains very small (Fritz et al., 2015; Gangopadhyay et al., 2022) which might explain the weak effect (or absence of an effect in Palearctic migrants) of field size impact on bird populations. Due to this uncertainty, the potential associations between field size (i.e., configuration) and species richness or abundance during winter should be interpreted with greater caution than those for composition.

There are several caveats to our study. We visited each cell across the survey grid in the study region only once due to financial, logistic, and time constraints. Therefore, we could not account for variation in phenology, especially for Palearctic species that arrive at different times during the wintering months (see Appendix S1: Figure S1). Due to the single‐visit surveys and the limited sample size for most species, we were unable to use distance sampling methods or occupancy modeling that accounts for variation in detection probability. Our bird data consisted of 26 Palearctic migratory species that were observed primarily around the agriculture and open‐ecosystems. They represent a small fraction of close to 470 migratory species that annually winter in India (BirdLife DataZone, 2024). Multi‐season‐multi‐visit surveys in the future could greatly improve our work to address winter associations between grasslands and avian populations in different parts of the Indian subcontinent. Open ecosystems, especially in western (Gujarat), northwestern (Rajasthan) India, and in Central Indian grasslands, might increase the number of Palearctic species that can be included in future assessments.

Due to high crop diversity during the winter season, small landholding size, and lack of validation data on individual crop types, we focused on a binary classification of annual winter (*Rabi*) crops and semi‐perennial crops, particularly targeting cash crops such as sugarcane. This approach is justified in our study region because of the very high proportion of sugarcane cultivated (Adhale et al., 2019; Dingre, 2023; Monfreda et al., 2008) (see Appendix S1: Figure S8). Fields with a long growing period of sugarcane differ strongly from the fields with annual crops with shorter growing periods and therefore have a lower cumulative primary productivity (INDVI) compared with consistently high values for sugarcane. However, given the diversity and high seasonality of annual and semi‐perennial crop types in other regions of the Indian subcontinent, we acknowledge that our INDVI thresholding method will need further validation and replication to assess its efficacy in other agricultural systems.

The wintering grounds of Palearctic migrants in agriculture–savanna mosaics of the Indian subcontinent remain largely unprotected and highly vulnerable to change despite their high value in the annual migration cycle for hundreds of species traveling in the Asian flyways. This change might come in the form of cropland expansion into grassland and intensification of land use, both factors that contribute to reaching the Zero Hunger sustainable development goal. A further driver of change could be an increasing demand for biofuel in India and globally (Hinz et al., 2020). In the UN decade of ecosystem restoration (2021–2030), the conservation and restoration of open ecosystems such as the agriculture–savanna mosaics studied by us need urgent reevaluation. Given the long‐lasting legacies of land use and land cover change in Indian savannas, defining a baseline time period to which the ecosystem can be “restored” would be a challenging task. While calling for a reevaluation of agricultural landscapes, our study also underscores the land‐sharing potential of these ecosystems (Agger et al., 2024; Fischer et al., 2014) to argue that restoration of these landscapes will require ecologically and economically sustainable solutions.

Savanna grasslands on the Indian subcontinent host over 200 species of endemic plants, approximately half of which were only described in the past two decades (Nerlekar et al., 2022; Ratnam et al., 2016). Semi‐perennial crops like sugarcane may be economically more viable in the short term, but the sustainability of further expansion and intensification of sugarcane agriculture in the western Indian landscape is questionable in the changing climate, stochasticity of the monsoon, depleting water tables, and degrading soil quality (Ghosh et al., 2012; Singh, 2016). Potential stagnation or decline in sugarcane yield over the past two decades is likely due to inadequate soil and water resource management and already highlights the economic challenges in continued intensive production (Dingre, 2023; Upreti & Singh, 2017).

For long, agricultural development in western India has been characterized by the expansion of sugarcane, a heavily irrigated semi‐perennial cash crop that became the financial cornerstone of agriculture in the state of Maharashtra in the 1950s (Attwood & Attwood, 2019; Lee et al., 2020). The area used for sugarcane in our study region tripled between 1998 and 2020, largely on former grasslands (Tian et al., 2014). Today, this region is one of the key sugar producers globally. While emphasizing the high conservation value of agriculture–savanna mosaic landscapes, we advocate for curbing the further expansion of semi‐perennial cash crops such as sugarcane in favor of protecting the remaining savannas in the Indian subcontinent. Continued loss of savannas to agricultural intensification will further contribute to the loss of avian biodiversity, especially Palearctic migratory birds that spend a significant portion of their life cycle in the subcontinent.

Globally, agricultural fields are getting bigger with better technology and changing government policies (White & Roy, 2015). Yet, the smallholder agricultural system has persisted in many parts of India despite the advancements in irrigation systems and enhanced crop varieties (Lesiv et al., 2019; Smale et al., 2008). This has little effect on agricultural productivity as the agriculture sector still maintains an inverse field size—productivity relationship in India. Instead, productivity is driven by intensification, namely irrigation infrastructure and mechanization (Sampath, 1992; Wang et al., 2015). Agriculture–savanna mosaics in the Indian subcontinent are some of the most productive agricultural systems globally. India is one of the top producers of sugarcane, wheat, rice, milk, legumes, and cotton (Gangopadhyay et al., 2022; Press Information Bureau, Government of India, 2020). India has the largest global cattle population and the largest dairy industry in the world (FAO, 2021). The diverse pastoralist communities that contribute to this industry primarily rely on the agriculture–savanna mosaics. Our work demonstrates that resident and Palearctic migratory avian species benefit from the existing diversity in habitat niches. If the goal is to restore open ecosystems over the forthcoming decade, monitoring and maintenance of remaining savannas in the Indian subcontinent, fostering traditional annual crop varieties along with semi‐perennials, and prevention of unnatural afforestation in agriculture–savanna mosaics are urgently needed.

## CONFLICT OF INTEREST STATEMENT

The authors declare no conflicts of interest.

## Supporting information


Appendix S1:

## Data Availability

Data and code (Bhagwat et al., 2025) are available in Zenodo at https://doi.org/10.5281/zenodo.14743841.
