# Supplementary material for: Landscape composition drives winter bird assemblages in agriculture–savanna mosaics of western India
Source: Ecol Appl. 2025 Mar 6;35(2):e70016. doi: 10.1002/eap.70016 (PMC11885864; doi:10.1002/eap.70016)
Supplement: Supplementary file 1 — Appendix S1: [file EAP-35-e70016-s001.pdf]

Journal name: Ecological Applications

Manuscript type: Article

Special Feature: Applied Ecology in India

Manuscript Title: Landscape composition drives winter bird assemblages in agriculture-savanna mosaics of western India

Authors: Tejas Bhagwat, Philippe Rufin, Tobias Kuemmerle, Johannes Kamp

Supporting Information – Appendix S1

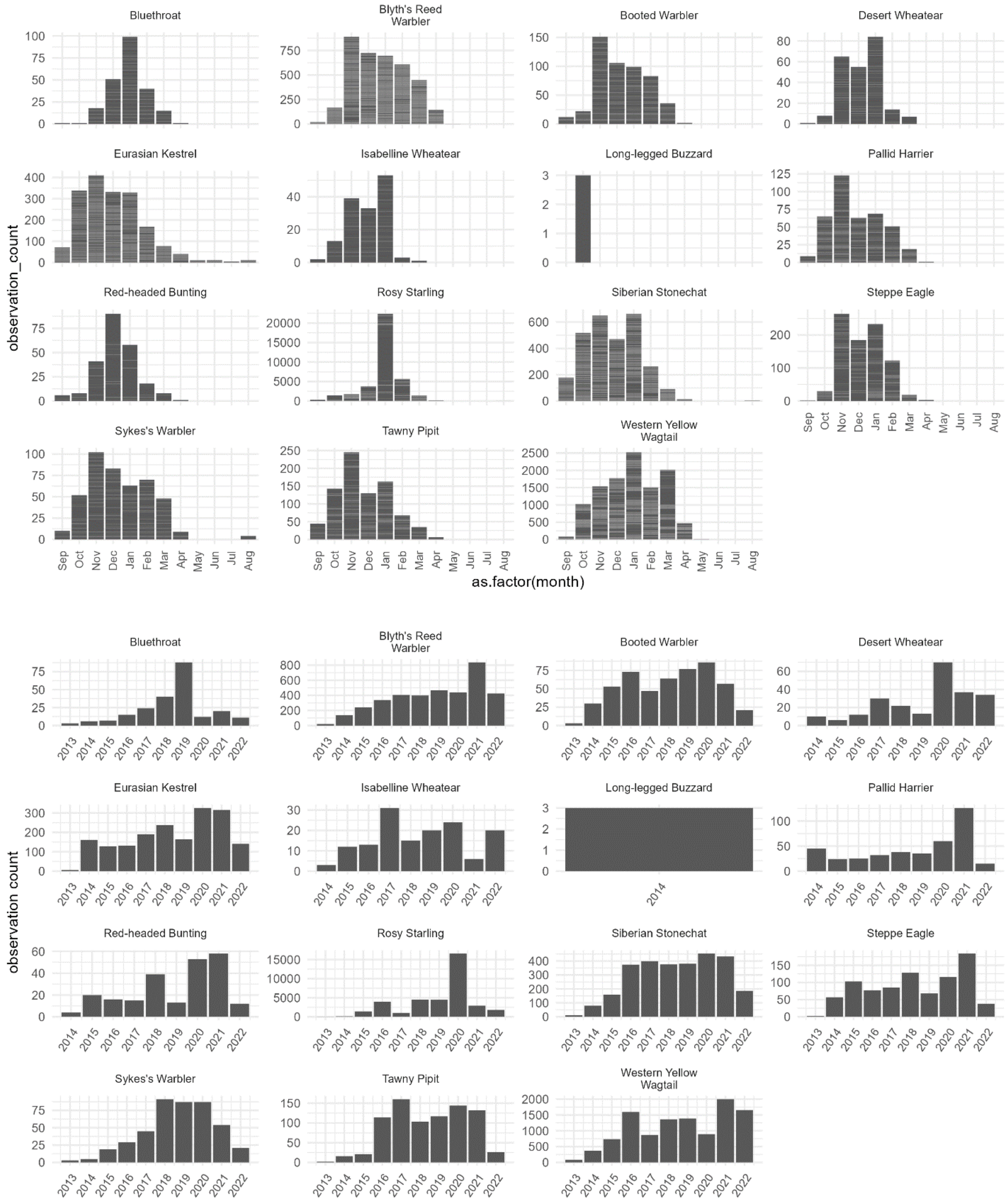

Figure S1**:** Distribution of Palearctic migrants across years and months in western Maharashtra (Source: eBird data)

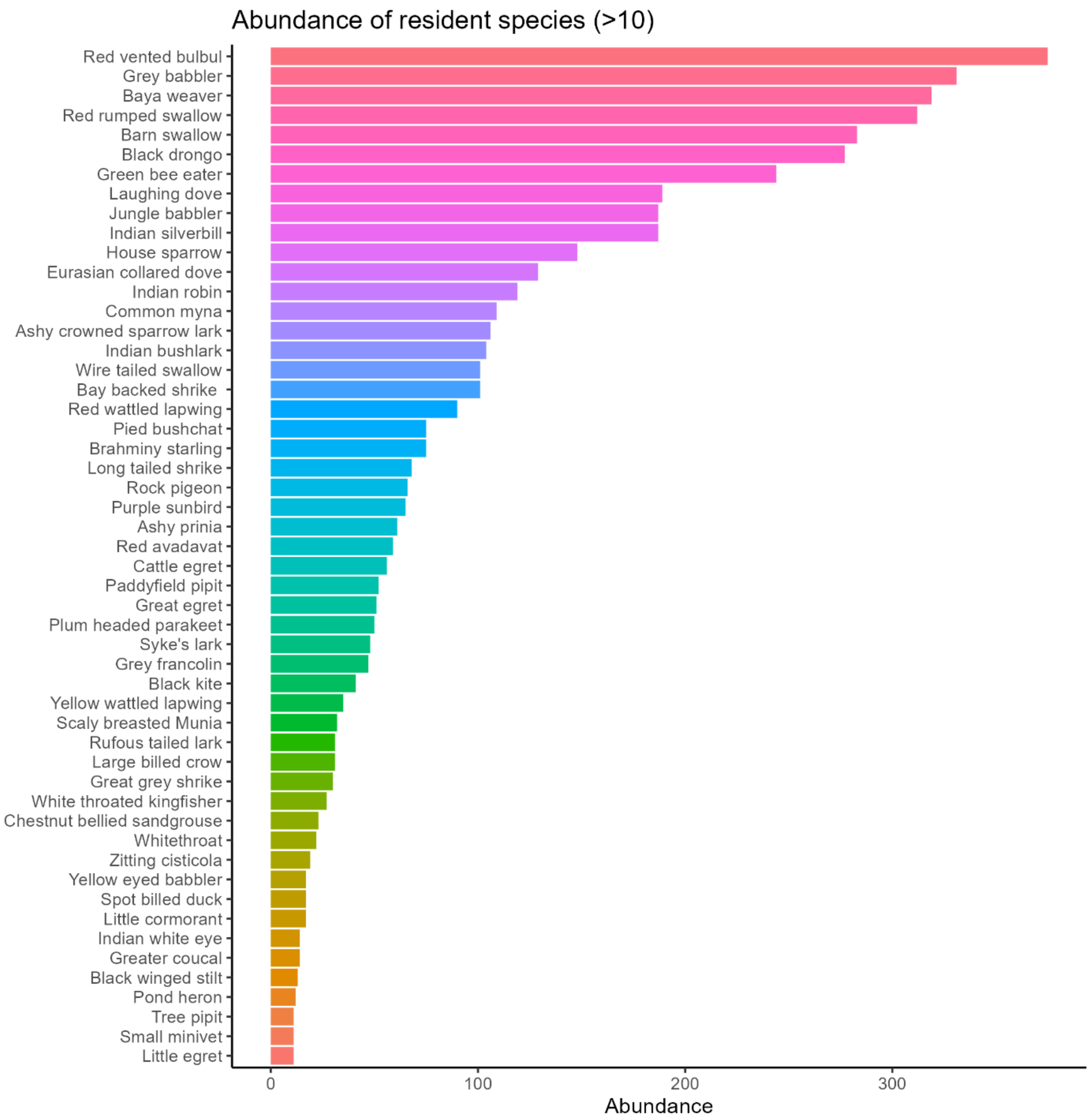

Figure S2**:** Total abundance of resident species (minimum 10 records) observed during the survey

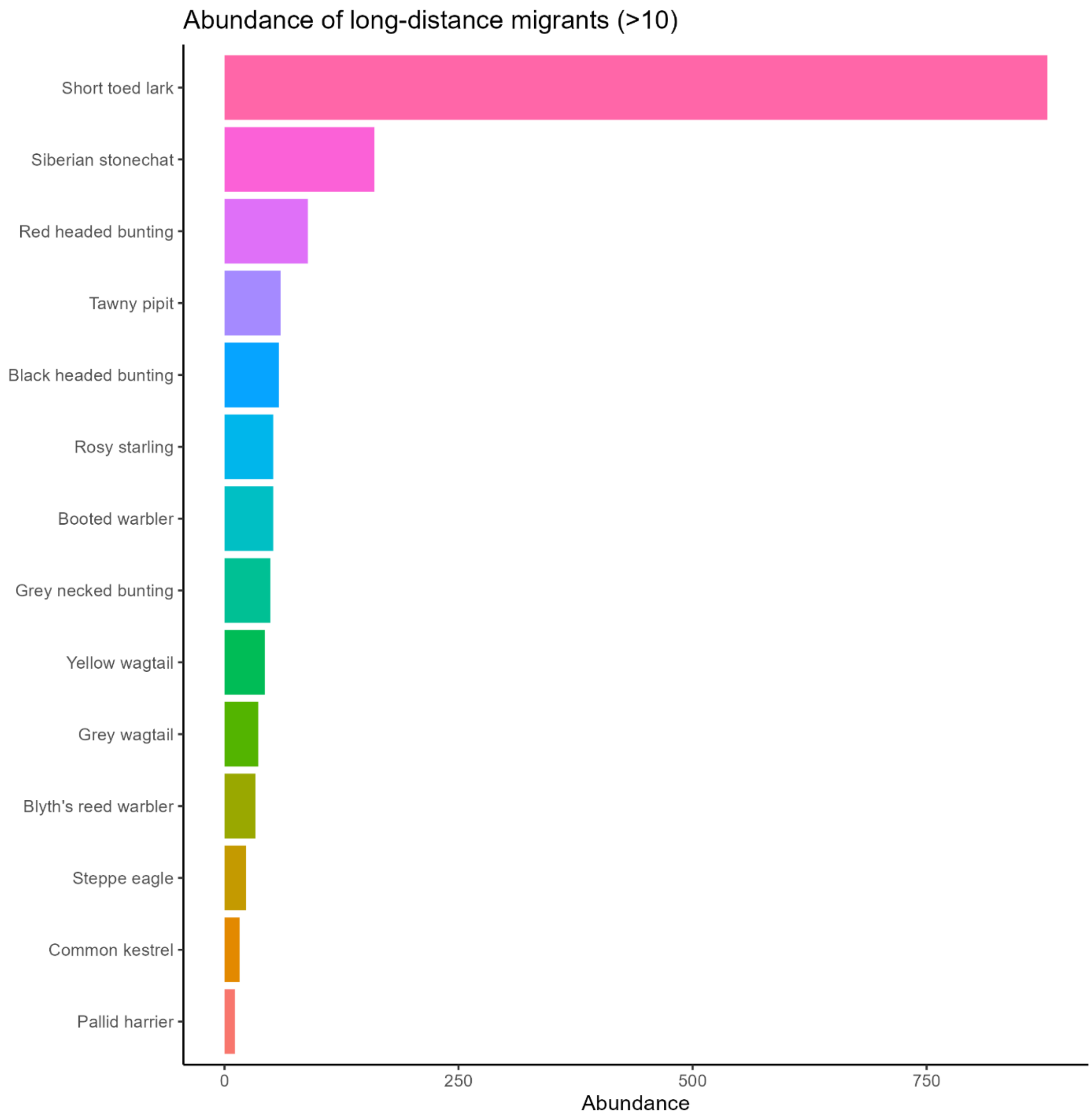

Figure S3: Total abundance of long-distance migratory species (minimum 10 records) observed during the survey

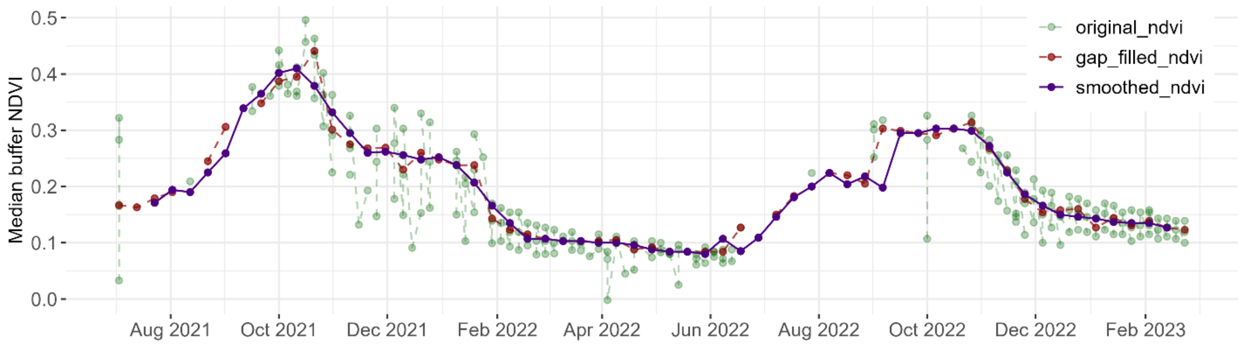

Figure S4: An example of NDVI profile for annual crops in Maharashtra. Raw Sentinel-2 NDVI values (Original NDVI), gap-filled NDVI values based on temporal unmasking to fill gaps left by cloud and cloud-shadow masks, and smoothed NDVI time-series based on Savitzky-Golay filter that removes remaining residual outliers in the data. For each pixel, we calculated INDVI as the sum of all NDVI values recorded over the 18-month period.

Table S1**:** Confusion matrix and catergory accuracies based on field information based accuracy assessment of land cover classification inside 150 m buffers surrounding the point count data. Producer’s accuracy (PA) refers to the proportion of a given reference land cover class that is mapped as given land cover class. User’s accuracy (UA) refers to the proportion of a mapped land cover class that has the given reference land cover class.

	**Reference**				
**Prediction**	Semi-perennial crops	Annual crops	Woody vegetation	Grassland open	**PA**
Semi-perennial crops	235	114	49	0	**0.73**
Annual crops	81	203	44	19	**0.63**
Woody vegetation	6	4	89	0	**0.38**
Grassland open	2	1	51	601	**0.97**
**UA**	**0.59**	**0.59**	**0.90**	**0.92**	

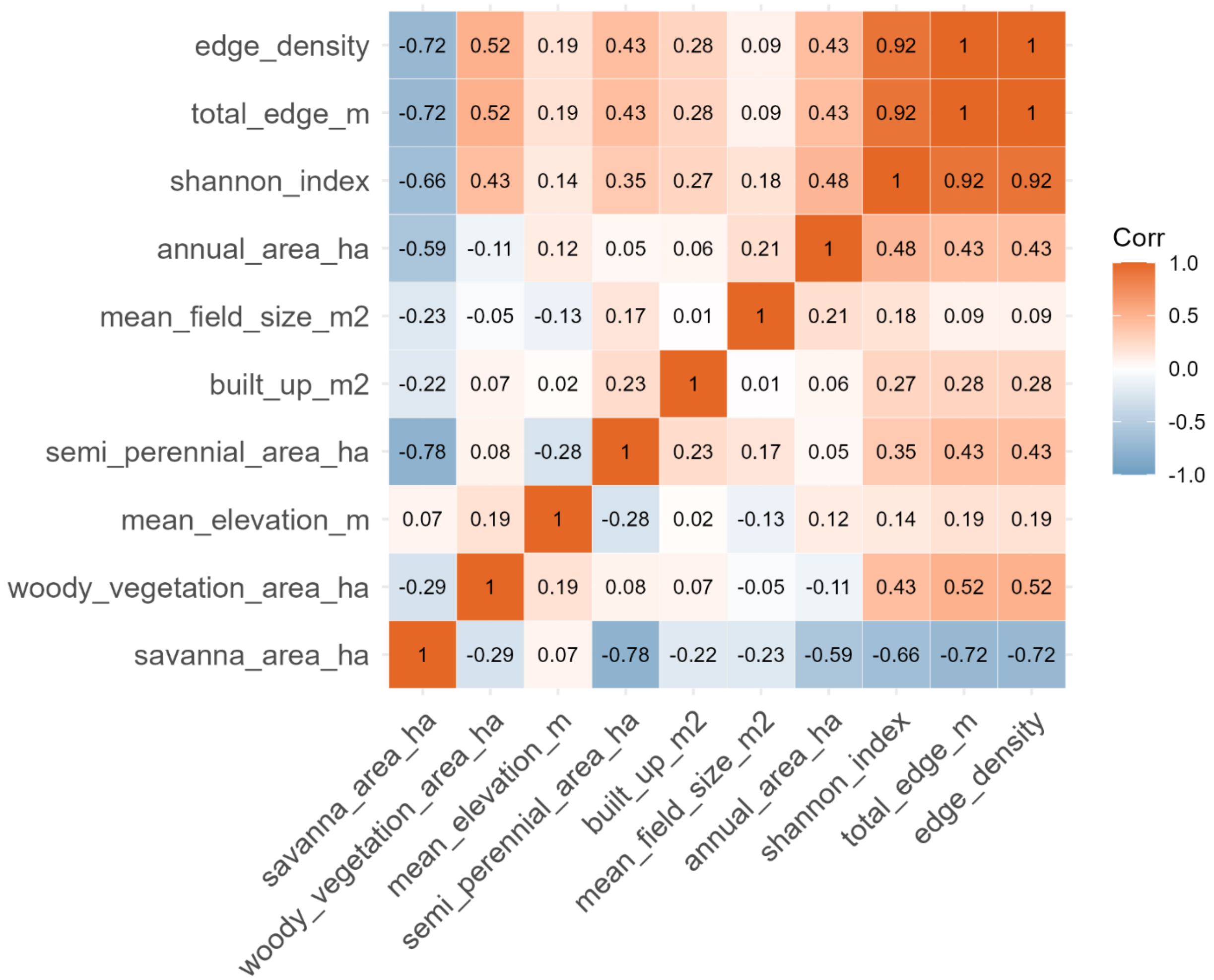

Figure S5: Spearman’s correlation coefficient for all the compositional and configurational parameters calculated for the study region

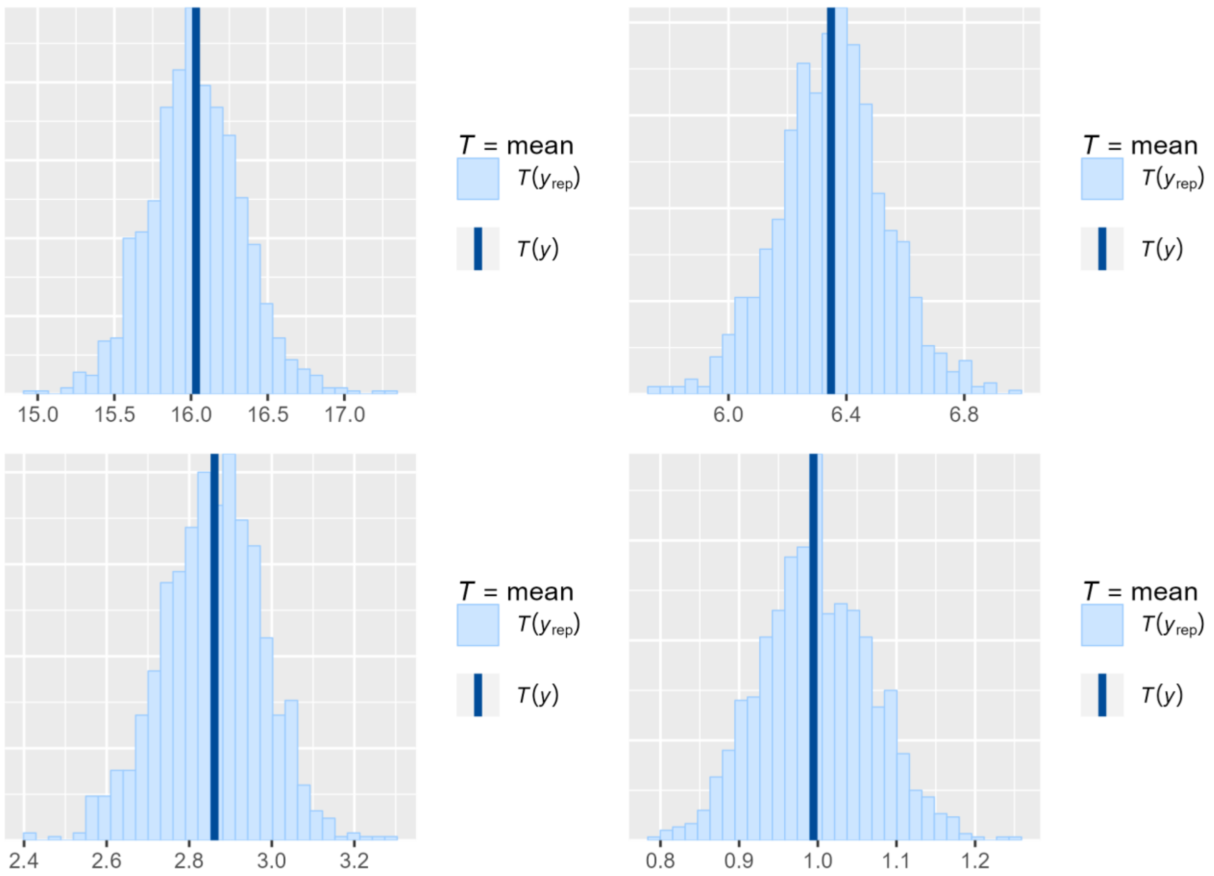

Figure S6: Models for resident bird abundance, species richness (top), and abundance Palearctic of migratory birds and species richness (bottom). Histograms show the frequency distribution for replicated data simulated under the model. Blue lines show the posterior means of the Chi-square test statistic for actual (observed) data.

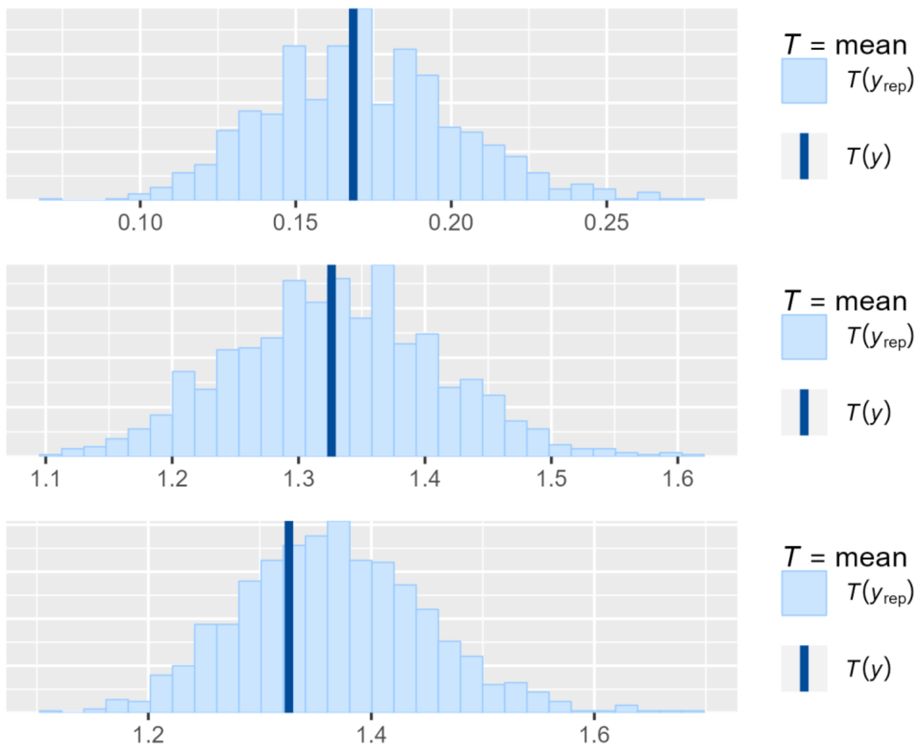

Figure S7: Models for guild based abundance (From top to bottom - Carnivores, open-ground dwellers and species preferring shurbs) of long-distance migratory species. Histograms show the frequency distribution for replicated data simulated under the model. Blue lines show the posterior means of the Chi-square test statistic for actual (observed) data.

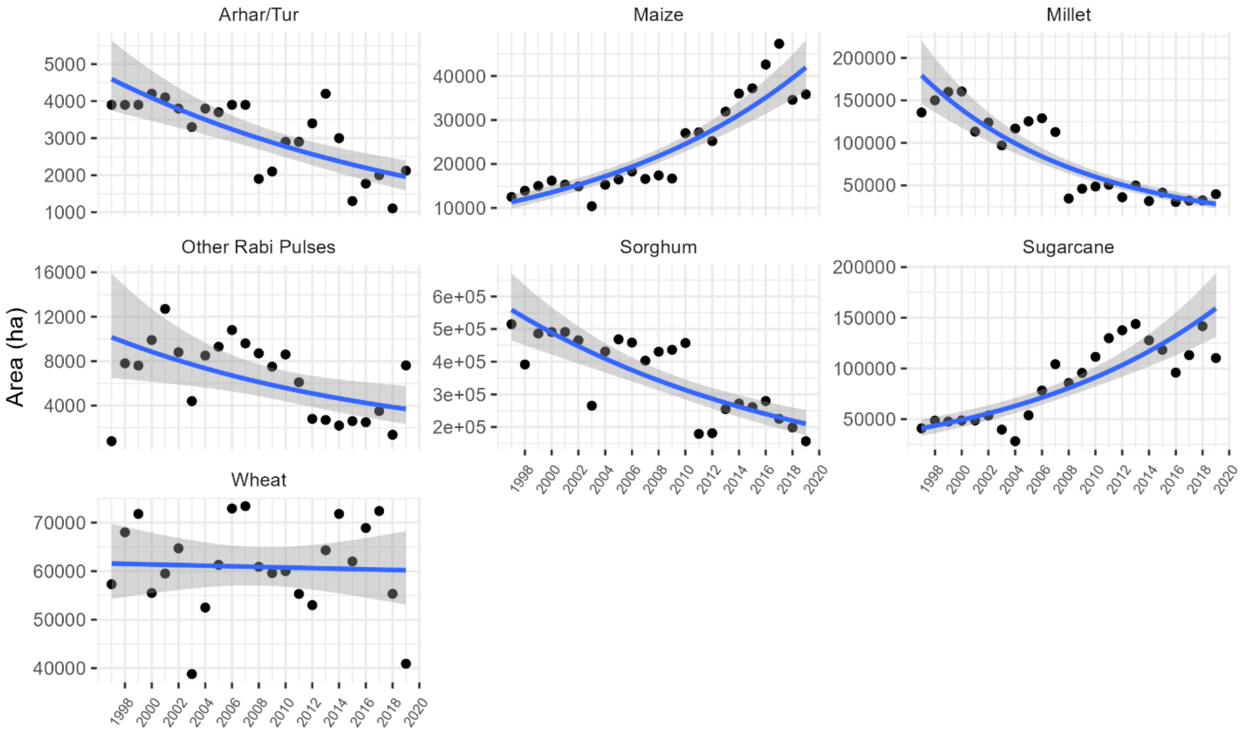

Figure S8: Cultivated area for the major crop types in Pune district, Maharashtra. The figure shows consistent growth in the area of annual crops namely sugarcane (all three varieties) over the past two decades, mirroring a decline in cultivation areas for other annual, traditional crop types during the same period. Figure produced by the authors based on the "District-wise, season-wise crop production statistics from 1997" available from the Open Government Data (OGD) Platform India at https://www.data.gov.in/catalog/district-wise-season-wise-crop-production-statistics-0.
